# The Foreign Journals: Medical Police

**Published:** 1841-04

**Authors:** 


### MEDICAL POLICE.

### *On Kyanised Timber.* By Dr. Schweig, of Carlsruhe.

The discovery made in England by Mr. Kyan, namely, that wood might be preserved from dry-rot and the destructive attacks of insects, has now, we are told by the author, come into such general use on the continent, as to require an examination into its influence on public health. Kyan's process consists in saturating wood thoroughly, by long immersion, with a solution of corrosive sublimate, in the proportion of two pounds of sublimate to one hundred measures of water.

In regard to the influence of this process on public health, the following points require attention:

1st. From the ready absorption of this poisonous liquid through the skin, the mere manipulation of the timber exposes the workmen to much danger.

They should be warned not to dip their hands in the liquid more than can be avoided. They should wear linen which ought to be frequently washed; the water used in washing either their persons or their linen should have muriate of ammonia dissolved in it, so as to precipitate and separate the poison more effectually. Lastly, the earth on which any of the poisonous liquid may fall should after a time be dug up and buried in a deep hole.

2d. As a matter of medical police, it is proper to enquire whether under certain circumstances mercury may not be volatilized from wood thus prepared, and produce its usual dangerous effects upon those exposed to the vapour.

The occurrence of prejudicial effects from this cause has been denied, because the crews of vessels built of Kyanised timber have returned healthy after long voyages, even in tropical latitudes. Where there is a free access of air, as in the wood used in railways (sleepers), no danger from this source is to be apprehended; but when the wood is employed in the construction of close apartments, in damp or ill-ventilated places the case is different. Experience may not have hitherto shown that dangerous consequences have followed; but if there be reason to suspect the possible occurrence of these on physical grounds, this is sufficient to justify a government in interfering to prevent the use of such timber for the construction of houses.

3d. It need hardly be said that wood thus impregnated with corrosive sublimade, is wholly unfitted for the making of vessels to hold articles of food or drink, either for the use of man or animals.

4th. A fourth and very important consideration is, that Kyanised wood when used up cannot be safely employed for fuel like ordinary wood; the mercury contained in old wood might be thus volatilized and spread in vapour through the apartments of a house, leading to the slow destruction of life by poison, by giving rise to protracted illness. It could only be safely burnt in close stoves or vessels, where there was a free current of air to carry off the mercurial vapour.

The chips or cuttings of wood of this description, might unknowingly be employed as fuel by labourers and others engaged in working the timber.

From the preceding remarks it may be inferred, that some precautions should be taken by a government in allowing the application of this process; and it is at the same time a duty to ascertain whether some harmless substitute equally preservative may not be found for the deadly drug at present used. The author says that a substitute of this kind is now well known and much employed, namely, the sulphate of copper. This effects the same chemical changes on the soluble principles of wood as corrosive sublimate; and although being a poison the wood cannot be used for the manufacture of domestic utensils, yet the metal copper is fixed, and when the wood after use is burnt, there is no fear of its giving out poisonous vapours. Owing to this it might also be safely employed in the construction of houses or in buildings for the use of man and animals. It is then a fair question whether a government ought not to prohibit the use of timber thus saturated with sublimate, and recommending as a substitute a solution of the sulphate of copper.

[We consider this to be a very important paper, and deserving the attention of a board of medical police, if there were such in this country. Fortunately the process in England is patented, and this circumstance has, to the great benefit of the community, restricted its application. Should the use of Kyanised timber spread after the expiry of the patent, which we believe there is little fear of, as the process is likely to be superseded by a more effective one, we may expect that cases of poisoning will be frequent, not so much in consequence of the fragments of wood being used for fuel, as from its being ignorantly used in the construction of barrels, tubs, cisterns, spouts for the holding of water, milk, wine, or other liquids. It is absurd to say that its use should be restricted to out-of-door purposes; since you cannot provide knowledge for the lower classes of artificers; and whether by accident or through ignorance, Kyanised wood will be frequently used for purposes not contemplated by the inventor of the process. The bare possibility of a danger of this sort occurring is, as Dr. Schweig observes, at once a sufficient justification for a government to interfere.

We believe that the author is mistaken in thinking that sulphate of copper is equally effective as corrosive sublimate, imperfect as even this is, and he seems quite ignorant of the more recent discovery of Sir William Burnett, that *chloride of zinc* is greatly superior to both in the preservation of wood, and yet more in the preservation of sail-cloth and cordage. The great superiority of this agent is yet further enhanced by its want of poisonous qualities. We believe Sir William Burnett has obtained a patent for this discovery, and that it is deservedly taking the place in this country of the more dangerous process of Mr. Kyan.]

*Henke's Zeitschrift, f. d. S.A.*; 1, 1840.

